# The UK National screening committee, the newborn genomes programme, and the ethical conundrum for UK newborn screening

**DOI:** 10.1007/s12687-025-00788-1

**Published:** 2025-06-11

**Authors:** Sara M. Rankin, Lucy Marskell, Lina Hamad, Laura Machin

**Affiliations:** 1 National Heart and Lung Institute, Imperial College London, London, UK; 2 Lancaster Medical School, Lancaster University, Lancaster, UK; 3 Faculty of Medicine, Imperial College London, London, UK; 4 rm 352 IRD section, NHLI, Faculty of Medicine, Imperial College London, London, UK

**Keywords:** Newborn screening, Genomic sequencing, Newborn screening policy

## Abstract

Countries in the Global North use biochemical tests to screen for at least 20 diseases in newborns, while in the UK, only 10 diseases are screened for. The United Kingdom National Screening Committee (UKNSC) is the entity responsible for making recommendations to the government with regards to which conditions should be included in the Newborn Screening (NBS) programme. Examination of the meeting minutes of the UKNSC between 2015 and 2022 revealed that no new diseases were recommended for NBS during this period. If there was no ‘effective treatment’ for the disease it was rejected for NBS. In 2022, the Newborn Genomes Programme (NGP) was announced; a research study aiming to screen for over 223 rare genetic diseases using whole genome sequencing technology in newborns. While this could lead to a seismic expansion of NBS in the UK, many of the diseases included in the programme are currently considered ‘actionable’ rather than ‘treatable’ conditions. This poses an ethical conundrum for the UKNSC, which is involved in both NBS and NGP, given that it has thus far made recommendations against the expansion of the NBS programme using available biochemical assays, contrary to what has been implemented in other countries in the Global North. In this paper, we aim to critically examine the processes and circumstances that have held back the expansion of the NBS programme in the UK, as compared with other countries, focusing on the period 2015–2022, when no new diseases were added to the UK NBS programme, and contrast them with the drivers that have led to the support and funding for the NGP during this same time.

## Introduction

There are over 7000 diseases, that are classified as rare genetic diseases. These are diseases that affect 1 or less people in every 2000 of the population. However, because there are thousands of different rare diseases, it is estimated that 1 in 17 people in the UK have a rare disease, this is equivalent to over 3.5 million people (UK Health Security Agency 2018; Genetic Alliance 2019). 75% of people affected by a rare genetic disease are children, with more than 30% of these children dying before their fifth birthday (Genetic Alliance 2019). Living with a rare genetic disease very often requires complex care and can be life limiting for the individual but also have a significant impact on carers in terms of financial stability and mental health (United Kingdom National Screening Committee 2019; European Organisation for Rare Diseases 2017; Delaye et al. 2022).

**Fig. 1:**
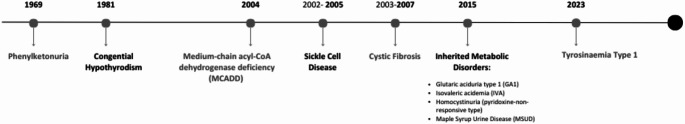
Conditions currently included in the United Kingdom newborn screening programme

In 1969 Newborn Screening (NBS) for phenylketonuria was introduced in the UK, in what was considered one of the earliest and most ground-breaking public health initiatives established to identify and manage rare diseases in infants shortly after birth (Downing and Pollitt 2008). In the UK, NBS primarily involves biochemical analysis of blood spots collected via heel-prick on day five after birth and has been extended to 10 diseases since 1969 (Fig. 1). The introduction of tandem mass spectrometry (MS/MS) in the 1990s provided the opportunity to screen blood spots for approximately 60 metabolites related to 50 different diseases in a cost-effective way (Carlie Driscoll and McPherson 2010). Since the introduction of this technology, many countries have expanded their NBS programmes. For example, the United States now includes 35 core conditions, Italy screens for 40 conditions, Australia includes 25 conditions, and both Japan and New Zealand screen for 20 conditions. In contrast, the UK currently screens for 10 conditions (Therrell et al. 2024).

While the UK was an early adopter of NBS, it has not expanded its NBS programme at the same rate as other countries in the Global North. Consequently, the UK has been criticised by many experts including paediatricians, obstetricians and clinical geneticists, as lagging behind.

The United Kingdom National Screening Committee (UKNSC) is the entity responsible for making recommendations to Government ministers and NHS Chief Medical Officers (CMOs) with regards to which conditions should be included in the NBS programme. They are the gatekeepers when it comes to expanding the NBS programme. Genetic Alliance UK (GAUK) - a group of scientists, clinicians, and charities for rare genetic diseases – criticised the approach taken by the UKNSC in relation to the NBS programme in a report published in 2019 (Genetic Alliance 2019). Criticism stems from considerable frustration that NBS for many rare metabolic diseases has been held back, when cost-effective screening tests exist. GAUK has argued that this has prevented many patients with rare genetic diseases from obtaining an early diagnosis over several decades.

Recent developments in “low-cost” genomic sequencing technologies provide an alternative methodology to identify rare genetic diseases. While they are considered ‘low-cost” in relation to the cost of sequencing technology a decade ago, they are still prohibitively expensive for UK-wide NBS. However, a more targeted application of these sequencing technologies has been assessed in critically unwell babies and children; with many studies demonstrating its effectiveness in establishing diagnoses and influencing clinical management in this population (Group et al. 2021; Mestek-Boukhibar et al. 2018; Chung et al. 2020; Dimmock et al. 2020; Horton and Lucassen 2023). Consequently, under the new Wales Infants and children’s Genome Service (WINGS), NHS Wales became the first country in the UK to introduce whole genome sequencing to rapidly diagnose rare diseases in critically ill babies and children (All Wales Medical Genomics Service 2019). In the first two years of the WINGS service, pathogenic or likely pathogenic variants were identified in 17 children from 45 families tested (Murch & Halstead 2021). The service has demonstrated significant health benefits for these patients, including changes to clinical management. (Jezkova et al. 2022; Murch & Halstead 2021). A similar programme in California showed the huge cost benefit of early diagnosis of critically ill children with rare genetic diseases, leading to the introduction of the “Ending the Diagnostic Odyssey Act 2021”. As a result, all 50 States’ Medicaid programmes now cover the cost of whole genome sequencing for critically ill children (Jezkova et al. 2022).

In 2022, the UK government announced the launch of the Newborn Genomes Programme (NGP), a project costing £105 million, which aims to sequence the genomes of 100,000 newborns, as part of an NHS-embedded study, for genetic conditions that may impact their health in the early years of life (Genomics England 2023c). The NGP is led by Genomics England, which was originally set up in 2013 by the United Kingdom Department of Health and Social Care to deliver the 100,000 Genomes Project (Genomics England 2025). While the UKNSC is not a direct partner in delivering the NGP, it has been involved in its development. In 2021, the UKNSC and Genomics England jointly commissioned a public dialogue to explore the programme’s implications for newborns (Genomics England 2021; Pichini et al. 2022). Experts from UKNSC are also members of Genomics England’s Clinical Assurance Group, which aims is to provide assurances that appropriate care and treatment for each condition in the study is accessible across the NHS (Genomics England 2023b).

Communications on Genomics England’s website state that the results of this study “*will add to evidence that will inform future decisions on using whole genome sequencing to support newborn screening*” (Genomics England 2023c). In October 2023, Genomics England published a list of 223 individual genetic conditions that will be included in the NGP (Genomics England 2023a). Many of the listed diseases have been previously rejected by the UKNSC from inclusion in the NBS programme. This creates a contradictory position for the UKNSC; if the primary goal of the NGP is to expand NBS, the UK could achieve this by extending the biochemical analysis of blood spots similar to other countries in the Global North. The NGP also raises many questions on the scope of the programme, informed consent and interpretation of uncertain findings (The Lancet 2023; Page 2023; Horton and Lucassen 2023). In this paper, we aim to critically examine the processes and circumstances that have held back the expansion of the NBS programme in the UK, as compared with other countries, as well as briefly consider the ethical aspects of the NGP. Specifically, we will focus on the period 2015–2022 when no new diseases were added to the UK NBS programme and contrast them with the drivers that have led to the support and funding for the NGP during this same time.

## Methods

A review of the literature was conducted focusing on NBS policy in the UK. This included a review of the relevant grey literature such as blogs published on the UKNSC official website, evidence maps conducted by commissioned external consultants published on the UKNSC website, and reports published by relevant organisations and rare disease patient advocacy groups such as Genetic Alliance UK and the European Organisation for Rare Diseases (EURORDIS). A critical analysis of the meeting minutes published on the UKNSC website was conducted for the period 2015–2022. Meeting minutes were reviewed and analysed to plot key points in the evolution of NBS policy, map the diseases submitted for screening recommendations, identify the most common reasons for disease rejection as per UKNSC criteria and construct a case study to demonstrate the current recommendation process for NBS in the UK and its outcomes in comparison to other European countries and the US. To note, the UKNSC meeting minutes are only available publicly from 2015. Meeting minutes prior to this date have not been published.

## Results

### The United Kingdom National screening committee and the evolution of the UK screening criteria

Established in 1996, the UKNSC serves to advise the NHS and ministers in all four countries of the UK with regards to all aspects of population screening and has responsibility for making recommendations with respect to which conditions are included in the screening programme. The UKNSC is accountable to the four CMOs, and currently recommends screening for 10 conditions via dried blood spots collected by a nurse, midwife or health visitor, five days following birth and sent to one of thirteen laboratories in the UK for testing (United Kindgom Government 2022b).

The conditions included in the UK NBS programme have been determined based on a set of criteria, derived from the principles originally developed by Wilson and Jungner in 1966 for general population screening (Table 1) (United Kingdom National Screening Committee 2022; Jungner G and Wilson JMG, 1966). The criteria have evolved since the establishment of the UKNSC in 1996, with a revised list of 20 criteria published two years later by the UKNSC in their first report (Table 1) (United Kingdom National Screening Committee 1998). Whilst the same set of screening criteria is currently in use, the process shifted in 2015, with annual calls put out for proposals to screen specific diseases (United Kingdom National Screening Committee 2023a). Valid proposals are taken forward by commissioning an evidence map from an external consultant (e.g. Costello Medical), whereby published research related to a particular proposed disease is reviewed against the 20 criteria set by the UKNSC to recommend screening. There is also public consultation, and anyone can submit a response to the call, including learned scientific or medical societies and individuals such as medical experts, scientists, patients, carers, and parents.

**Table 1:** Comparison of National screening committee criteria for population screening programme with original Wilson and Jungner principles of disease screening

Category	National Screening Committee Criterion	Description	Original Wilson and Jungner principles of disease screening
The Condition	**1**	The condition should be an important health problem.	The condition sought should be an important health problem.
	**2**	The epidemiology and natural history of the condition should be understood, with detectable risk factors or disease markers and a latent or early symptomatic stage.	There should be a recognisable latent or early symptomatic stage.
			The natural history of the condition, including development from latent to declared disease, should be adequately understood.
	**3**	Cost-effective primary prevention interventions should be implemented where practicable.	
The Test	**4**	*There should be a simple, safe, precise, and validated screening test.	There should be a suitable test or examination.
	**5**	The distribution of test values in the target population should be known, and a suitable cut-off level defined and agreed upon.	
	**6**	The screening test should be acceptable to the population.	The test should be acceptable to the population.
	**7**	*There should be an agreed policy on further diagnostic investigation for individuals with a positive test result and treatment choices.	There should be an agreed policy on whom to treat as patients.
The Treatment	**8**	*There should be an effective treatment or intervention for patients identified through early detection, with evidence of better outcomes with early treatment.	There should be an accepted treatment for patients with recognised disease.
	**9**	Evidence-based policies should cover which individuals should be offered treatment and the appropriate treatment to be offered.	
	**10**	Clinical management and patient outcomes should be optimised by healthcare providers before the screening programme.	
The Screening Program	**11**	Evidence from high-quality trials should show that the screening programme effectively reduces mortality or morbidity.	
	**12**	The complete screening programme should be clinically, socially, and ethically acceptable to health professionals and the public.	
	**13**	The benefits of the screening programme should outweigh the physical and psychological harm caused by testing, diagnosis, and treatment.	
	**14**	The opportunity cost of the screening programme should be economically balanced in relation to overall medical care expenditure.	The cost of case-finding (including diagnosis and treatment of patients diagnosed) should be economically balanced in relation to possible expenditure on medical care as a whole.
	**15**	Clinical management of the condition and patient outcomes should be optimised in all health care providers prior to participation in a screening programme.	
	**16**	There must be a plan for managing and monitoring the screening programme with agreed quality assurance standards.	
	**17**	*Adequate staffing and facilities for testing, diagnosis, treatment, and programme management should be available before the screening programme starts.	Facilities for diagnosis and treatment should be available.
	**18**	All other options for managing the condition should be considered to ensure cost-effective interventions are in place.	
	**19**	Evidence-based information explaining the consequences of testing, investigation, and treatment should be provided to potential participants.	
	**20**	Anticipate public pressure for widening eligibility criteria, reducing the screening interval, and increasing testing sensitivity. Decisions should be scientifically justifiable to the public.	
			Case-finding should be a continuing process and not a “once and for all” project.

*Indicating when an original Wilson and Jungner principle has been modified in the National Screening Committee criteria

Our analysis of meeting minutes and evidence maps of the 20 diseases put forward to the UKNSC reveals that none were recommended for NBS between 2015 and 2022. Tyrisonaemia Type 1 was recommended in early 2023 and only Severe Combined Immunodeficiency Disorder (SCID) has progressed to a pilot screen (Mackie 2023). Table 2 provides the list of the UKNSC criteria (from those cited in Table 1) not met for each of these 20 diseases. The three most common reasons for the UKNSC not recommending NBS for a specific disease are lack of a specific test (cited in seven cases), lack of high-quality randomised-controlled trials showing that the screening programme is effective in reducing mortality or morbidity (cited in five cases), and lack of UK-specific prevalence data (cited in five cases).

**Table 2:** Diseases reviewed for newborn screening in the UK since 2015

Date	Disease	Criteria not met*	Recommended for screening YES/ NO
Nov 2015	Congenital Adrenal Hyperplasia (CAH)	4	NO
Nov 2015	Mucopolysaccharidosis I (MPS I)	1,4,6,8 no UK data	NO
Nov 2015	Neuroblastoma	11	NO
Nov 2016	Organic Acid Oxidation Disorder (PA & MMA)	8?, 11	NO
Nov 2016	Familial Hypercholesterolaemia	6,7,9,10,11,12	NO
June 2016	SCID		In discussion
June 2016	Kernicterus (previously evaluated 2011)	4	NO
June 2016	Krabbe Disease	Lack of peer reviewed evidence	NO
June 2017	Thrombophilia	4,9	NO
June 2017	Tyrosinaemia Type 1	1 The impact false negative test results have on babies is unknown	NO
June 2017 / Oct 2017	SCID		In discussion
Oct 2017	Biliary Atresia	4, 8	NO
Feb 2018	Biotinidase deficiency (Previously reviewed in 2012)	1, 5, (11) no UK data	NO
Feb 2018	Spinal Muscular Atrophy (SMA)	1,4,9,10,11	NO
Feb 2019	Gaucher Disease	9	NO
June 2019	Long-chain 3-hydroxyacyl dehydrogenase (LCHAD) deficiency and Mitochondrial Trifunctional Protein (MTP)	1,4,9,11	NO
Feb 2020	Mucopolysaccharidosis type I (MPS I)	4,9	NO
Oct 2020	Galactosaemia	4,8	NO
Sept 2021	SCID		2 year Evaluation rolled out in Sept 2021
Nov 2021	Adrenal hyperplasia	4	NO
Nov 2021	Duchenne Muscular Dystrophy	4	NO
March 2022	Biotinidase deficiency	1,4- no UK specific data	NO
March 2022	Cytomegalovirus	4,8	NO

*UKNSC criteria number as in Table 1

### Comparison of newborn screening criteria in the United States, the United Kingdom, and other European countries

In 2003, the Advisory Committee on Heritable Disorders in Newborns and Children (ACHDNC) was formed to advise the Secretary of Health and Human Services (SHHS) about newborn and childhood screening. In 2004, the ACHDNC reviewed the panel of conditions recommended for national implementation. The American College of Medical Genetics (ACMG, now the American College of Medical Genetics and Genomics) was tasked with collecting expert opinions and analysing scientific literature on NBS (Health Resources and Services Administration - Advisory Committee on Heritable Disorders in Newborns and Children, 2023). These findings were intended to inform recommendations, including the establishment of a standardised panel of conditions. The panel was finalised in 2005 and subsequently recommended to the SHHS, which officially approved it in 2008 (Health Resources and Services Administration - Advisory Committee on Heritable Disorders in Newborns and Children, 2023). The initial Recommended Uniform Screening Panel (RUSP) included 29 core conditions and 25 secondary conditions. Core conditions were those deemed suitable for immediate implementation, while secondary conditions were those that could be detected during screening for a core condition but required further research due to insufficient scoring. In 2010, severe combined immunodeficiency (SCID) was added, and by 2016, the panel had expanded to 35 core conditions and 26 secondary conditions (Health Resources and Services Administration - Advisory Committee on Heritable Disorders in Newborns and Children, 2023).

The ACHDNC follows a structured, evidence-based approach for evaluating conditions nominated for inclusion in the RUSP. After a condition gets nominated by researchers or advocacy groups, an external group compiles and analyses data for the ACHDNC, drawing from systematic literature reviews, decision-analytic modelling, and stakeholder input (Goldenberg et al. 2016). This process is structured around the chain of evidence, encompassing newborn screening, follow-up diagnostics, and treatment outcomes (Goldenberg et al. 2016). The ACHDNC then evaluates the net benefit of screening based on health outcomes, benefits, harms, and screening effectiveness, assigning a rating from A (high benefit) to L (low certainty of benefit) (Kemper et al. 2014). In 2013, the decision-making process was revised to include an assessment of the capability of newborn screening programmes to implement the test, evaluating feasibility and readiness (Kemper et al. 2014). The Decision Matrix integrates these ratings to guide recommendations, with conditions rated A1 or A2 being strongly recommended, while others may require further research or system improvements (Kemper et al. 2014). The final decision is submitted to the SHHS, who provides guidance for state-level implementation (Table 3).

**Table 3:** Decision-Making process for conditions nominated to the recommended uniform screening panel (RUSP)

Step	Description
Nomination	A condition is proposed for inclusion in the RUSP by researchers, advocacy groups, or other stakeholders.
Evidence Review	An external evidence review group gathers data on screening benefits and harms from published and unpublished sources.
Evidence Report	A systematic review and decision analytic model is externally conducted to estimate potential benefits and risks of screening.
Assessment of Net Benefit	The ACHDNC assigns a rating based on health outcomes, treatment benefits, and potential harms: (A) High certainty of significant benefit. (B) Moderate certainty of significant benefit, but further research may refine findings. (C) Small to zero net benefit. (D) Negative net benefit, meaning screening could do more harm than good. (L) Low certainty due to insufficient evidence.
Assessment of Capability to Screen	Evaluates whether state newborn screening programs can implement testing, assigning: (1) Ready for implementation within a year. (2) Developmentally ready, but requires 1–3 years. (3) Feasible, but unprepared, requiring more than 3 years. (4) Low feasibility, making implementation impractical.
Decision Matrix Evaluation	Combines the Net Benefit and Capability Ratings to guide decisions: (A1) or (A2) Strong recommendation for inclusion. (A3) or (A4) Considered for inclusion, but improvements in readiness may be needed. (B), (C), (D), or (L) Not recommended, but future research may change eligibility.
Final Recommendation	The ACHDNC submits its recommendation to the Secretary of Health and Human Services.

Unlike a nationally mandated screening programme, the RUSP serves as a federal guideline for NBS. Individual states retain the authority to determine which conditions to include in their programmes. However, several states have enacted laws that align their NBS programmes with the RUSP, ensuring that any condition added to the federal panel is promptly included at the state level (Salova 2025).

Of note, in seven cases noted in Table 2, where the UKNSC did not recommend screening due to lack of a specific test, these diseases are currently screened for in the US programme (Biotinidase deficiency, Congenital Adrenal Hyperplasia, Galactosaemia, Long-chain 3-hydroxyacyl dehydrogenase deficiency, Mitochondrial Trifunctional Protein, Mucopolysaccharidosis I) (Health Resources and Services Administration - Advisory Committee on Heritable Disroders in Newborns and Children 2023). Indeed, eleven of the diseases not recommended by the UKNSC for newborns in UK are part of the NBS programme in the US, which highlights the dramatic difference in the approval process between the US and the UK. The UKNSC utilises this difference to claim that the UK process is more rigorous, whereas others argue that the UK screening criteria are not appropriate for all diseases, specifically rare genetic diseases (Genetic Alliance 2019; Page 2023; Downing and Pollitt 2008). It could be argued that the difference in the scope of the NBS programme between the US and the UK may be explained by variations in health economics and the contrast between public and private healthcare systems. However, the argument of cost versus benefit is not part of the criteria used by the UKNSC to make its initial recommendations to ministers and CMOs.

NBS programmes across European countries exhibit significant variability in both the number of conditions screened and the decision-making processes governing their inclusion. While some countries, such as the Netherlands (23 conditions) and Poland (29 conditions), have extensive screening panels, others, such as Greece (5 conditions) have more limited programmes (Therrell et al. 2024; Loeber et al. 2021). The governance over the screening policy also differs, with centralised bodies similar to the UKNSC such as Germany’s Federal Joint Committee and Netherland’s Centre for Population Screening of the National Institute for Public Health and the Environment overseeing inclusion based on predefined criteria, whereas countries like Italy and Spain allow regional health authorities to determine screening policies. Despite these differences, many countries in Europe still screen for considerably more conditions than the UK. For example, Sweden, Portugal and Austria screens for 24 conditions (Therrell et al. 2024; Loeber et al. 2021).

### Case study – biotinidase deficiency

Biotinidase deficiency (BD) is an autosomal recessive metabolic disorder that affects the BTD gene; this gene is responsible for producing an enzyme called biotinidase (Online Mendelian Inheritance in Man (OMIM), 2023). The disorder occurs due to an absence of biotinidase activity, which results in the body’s inability to breakdown and recycle biotin, a B vitamin (Online Mendelian Inheritance in Man (OMIM), 2023). In the absence of normal biotinidase activity, babies tend to develop primary neurologic symptoms such as seizures, hypotonia, vision problems and hearing loss, along with cutaneous abnormalities, including skin rashes, alopecia and recurrent viral or fungal infections (Chedrawi et al. 2008; Yang et al. 2020). Treatment consists of lifelong oral supplementation with unbound (free) biotin (Dahiphale et al. 2008). Children diagnosed before symptom manifestation generally remain asymptomatic and appear to have a normal development if adequate adherence to biotin supplementation is maintained (Dahiphale et al. 2008; Szymanska et al. 2015). If babies are not diagnosed and treatment is delayed, children suffer different degrees of irreversible neurologic symptoms such as hearing loss, visual abnormalities, and developmental delays (Liu et al. 2023).

The 2021 evidence map concludes that whole population screening for BD in newborns should not be introduced in the UK and that the current recommendation should be retained (Costello Medical., 2021). The justification for this decision was based on two observations; firstly, while some evidence on the prevalence and incidence of BD in high-income countries exists, currently there is no evidence on the prevalence and incidence rates of BD in the UK (Costello Medical., 2021). Secondly, while evidence is available on the accuracy of current screening tests using the dried blood spots for BD in high-income countries, no UK-specific evidence was found (Costello Medical., 2021). It was then established that the limited number of studies currently available, the heterogeneity in the index tests examined, and the lack of consistency in the outcomes reported limited the comparability of the evidence available (Costello Medical., 2021).

On the basis of this evidence map, the UKNSC concluded that the volume and type of evidence related to screening for BD is currently insufficient to justify an update review at this stage and should be reconsidered in three-years time. Thus, while the UK still does not screen for BD, it is screened for in over 30 other countries, including the US.

(Wolf et al. 1985; Costello Medical., 2021; Therrell et al. 2024). Importantly the decision, not to recommend screening for BD, moved forward despite consultation responses from the Royal College of Paediatrics and Child Health and University College London Great Ormond Street Institute of Child Health urging for early screening for BD and citing evidence on improved outcomes when early treatment is initiated (Wolf 1993; Costello Medical., 2021). Barry Wolf, the pioneer of BD newborn screening, published in 2017 on the successful long-term outcomes of adolescents and adults with profound BD who were identified through newborn screening, showing normal cognitive development, academic achievement, and healthy pregnancies in treated individuals (Wolf 2017). Interestingly, BD is in the recently published list of diseases to be included in the upcoming NGP, despite being rejected for NBS by the UKNSC in 2012, 2018 and 2022.

### Rare diseases and the voice of patients and parents

Rare diseases, though individually affecting only a limited number of patients, collectively impact a substantial portion of the global population. It is estimated that between three to six% of the global population suffer from a rare disease (Nguengang Wakap et al. 2020). Living with a rare disease presents a lifelong challenge, encompassing complex care needs that can significantly impact people’s quality of life (Ferreira 2019). Early diagnosis plays a pivotal role in providing individuals with rare diseases an opportunity to be involved in clinical trials and other research studies (United Kingdom Government Department of Health 2013; United Kingdom Government Department of Health 2023). Additionally, early diagnosis alleviates the emotional distress of families grappling with uncertainty, reduces the financial burden on the NHS by shortening an often-prolonged diagnostic odyssey, and facilitates the engagement of caregivers with patient support groups, offering invaluable enhancements to the quality of life for both the patients and their caregivers (Genetic Alliance 2019; European Organisation for Rare Diseases 2021). It is therefore vital that the voice of rare disease patients and that of their carers and family members is taken into consideration when developing wider national policy.

The report published in 2018 by GAUK was critical of the UKNSC with regards to how they had modified the original Wilson and Jungner criteria in a way that would make it highly unlikely to gain approval for NBS of a rare genetic disease (Genetic Alliance 2019). They noted that this had been done by re-wording of the original Wilson and Jungner criteria, for example “suitable test” had been changed to “validated test”, and “acceptable” treatment had been changed to “effective treatment” (Genetic Alliance 2019). Moreover, the inclusion of new criteria, such as the requirement for a high quality randomised controlled trial, created an additional barrier to the addition of rare genetic diseases to the UK NBS programme (Genetic Alliance 2019). In the UKNSC minutes published in 2020, it is noted that the committee reviewed the 2018 GAUK report and sent a response to the authors, but did not make any changes to the criteria with regards to NBS screening for rare genetic diseases (United Kingdom National Screening Committee, 2020). Similarly, no changes were made following the publication of EURORDIS recommendations in 2021 which promote screening that is proportionate to the reality of evidence challenges with rare diseases, and should not be unreasonable or impossible (European Organisation for Rare Diseases 2021). Nevertheless, in a blog on the website that celebrated 25 years of the UKNSC, the committee contended that the UK has “the most robust screening process in the world” (United Kingdom National Screening Committee 2021) (Table 4).

**Table 4:** The UK National screening committee four principles of ethical evaluation

Principle 1	Improve health and wellbeing
Principle 2	Treat people with respect
Principle 3	Promote equality and inclusion
Principle 4	Use public resources fairly and proportionately

### Reform of the United Kingdom National screening committee

With the reorganisation of the UKNSC, a Blood Spot Task Group (BSTG) was established in 2022 consisting of paediatricians, academics, ethicists, quality assurance professionals, geneticists, as well as patient and public voice representatives (United Kingdom Government 2022). The task group’s first aims are to compare the UK screening and implementation practices with the EURODIS key principles in NBS, develop recommendations that meet the challenges of finding good quality evidence on the accuracy of different tests for rare genetic diseases, and develop a publication on the challenges and solutions in economic models relating to NBS (Seedat F., 2022).

Review of the BSTG meeting minutes in July 2023 reveals that a manuscript comparing the EURORDIS principles with UK practices was submitted for peer review, taking into consideration feedback received from patient and public voice members (United Kingdom National Screening Committee 2023b). In the paper, which was published two months later (Lombardo et al. 2023), the UKNSC concluded that UK practices are only partially aligned with the EURORDIS first principle, which recommends identifying opportunities to support the newborn and their family as broadly as possible, including making recommendations for screening of actionable conditions - defined by EURORDIS as conditions where early intervention leads to health benefits for the newborn, conditions where facilitation of early diagnosis avoids a prolonged diagnostic odyssey, or where there are improved outcomes for the family such as access to patient groups and informed reproductive rights (European Organisation for Rare Diseases 2021).The UKNSC maintains that NBS should only be recommended when a disease is treatable (Lombardo et al. 2023), which is in contrast to the approach of EUDORIS. It is surprising, therefore, that Genomics England has taken the decision to identify 223 rare genetic diseases in babies, most of which are not treatable, but are considered actionable diseases (Genomics England 2023c).

During 2020 and 2021, the UKNSC worked with a representative of the Nuffield Council on Bioethics to review the way the committee considers the ethical aspects of the current screening programme, and new members with expertise in ethics and social science were recruited (Joynson 2021). This resulted in the suggestion of four new core ethical principles that should be considered in the decision-making process of the UKNSC when examining new cases for NBS (Table 5) (Joynson 2021). However, it is not clear from the UKNSC minutes whether consideration of these four new ethical principles has had any influence on the committee’s current decision-making process.

In summary, the minutes of the UKNSC between 2015 and 2022 show that the UKNSC has rigidly adhered to an algorithmic decision-making process, which requires each of the 20 screening criteria (Table 1) to be met before recommending a new disease for NBS. As a result, between 2015 and 2022 no new disease has been added to the UK NBS programme, despite the voice of parents, and medical and scientific experts. By contrast, other countries have expanded their screening programmes considerably during this time by using low-cost biochemical assays and adopting a more pragmatic approach to their screening criteria.

**Table 5:** Ethical principles guiding the selection of conditions included in the newborn genomes programme

	Ethical principle
1.	There is strong evidence that the genetic variant(s) causes the condition and can be reliably detected.
2.	A high proportion of individuals who have the genetic variant(s) would be expected to have symptoms that would have a debilitating impact on quality of life if left undiagnosed.
3.	Early or pre-symptomatic intervention for the condition has been shown to lead to substantially improved outcomes in children, compared to intervention after the onset of symptoms.
4.	Conditions screened for are only those for which the interventions are equitably accessible for all.

### Genomics England and the introduction of the newborn genomes programme

In 2016, CMO Dame Sally Davies entitled her annual report ‘Generation Genome’, setting the stage for establishing Genomics England and the 500,000 Genome Project, Genomic England’s first initiative to sequence adult patients affected by rare diseases or cancer (Davies C. S., 2016). The main argument put forward for this work is the potential of personalised medicine and prevention over cure, which is predicted to increase population health and reduce healthcare costs. The project was funded by the Wellcome Trust (an independent medical charity), UK Research and Innovation (UKRI) with four Biopharmaceutical companies (Amgen, Astra Zeneca, GSK, and Johnson & Johnson), each contributing £120,000 million to the project (Bell 2019). Of note, the genomic data of individuals participating in the project was linked to their healthcare data, which was provided by the NHS. The full anonymised data (genomic and healthcare) from the 500,000 genomes project was released by the UK Biobank in 2023 with the four BioPharmaceutical companies given early access to the data, nine months before it was made public (Bell 2019). The value of this resource to the scientific community and businesses (eg Biopharma, healthcare and health insurance) is immeasurable and data from the BioBank has already contributed to over 9000 scientific research papers (Callaway 2023).

In 2020, Genomics England announced a public dialogue, jointly commissioned by the UKNSC, to assess whether the public would support whole genome sequencing of 100,000 newborns (Hopkins Van Mil., 2021; Pichini et al. 2022). A total of 133 participants took part in the public dialogue and the responses were reported to be ‘largely positive” (Hopkins Van Mil., 2021). This report has been used to evidence the public’s approval of genomic screening of newborns. However, participants’ demographics data such age, gender, religion, ethnicity, and educational level was not made available in the report. This information is important for assessing the validity of the study, and its absence limits the ability to evaluate the generalisability of the findings. Moreover, the small sample size did not allow for stratification of opinions according to different characteristics e.g. pregnant women, parents etc. Nevertheless, based on the “largely positive” response from the public consultation, an independent ethics committee was established to determine the criteria for inclusion of genetic diseases in the NGP (Genomics England 2023d). In 2022, a public survey with respect to these criteria was undertaken and four ethical principles were identified to guide the choice of conditions to be screened for as part of the NGP (Table 5) (Genomics England 2023b).

These principles diverge significantly from the criteria set by the UKNSC with respect to NBS. Firstly, the language used to describe these principles is open to interpretation, in particular when determining what is considered “strong evidence” or a “high proportion” of individuals. Secondly, the third principle set by Genomics England does not specify requirement for UK specific data or evidence from double-blind randomised clinical trials, which are conditions that need to be met for UKNSC to recommend screening. Moreover, whilst Genomics England published the list of conditions that will be included in the NGP, the evidence maps showing how these conditions meet the ethical criteria have not been made publicly available (Genomics England 2023a). It is clear that the ethical principles guiding the choice of conditions the NGP aims to identify through whole genome sequencing differ significantly from those of the UKNSC. While we would expect to see a change in ethical principles with time, it would not be ethically and morally acceptable to have the UKNSC NBS programme and the NGP operating at the same time, given that NGP is being promoted on the Genomics England website as ‘an extension of the NBS programme’, giving the impression that diseases screened for in the NGP could become part of the NBS in the future (Genomics England 2023c).

It could be argued that Genomics England does not have to strictly adhere to the UKNSC criteria. However, the NGP is a study involving 100,000 newborns and their families, and is imbedded in, and jointly run by, the NHS. Horton and Lucassen provide a critical examination of the complexities and challenges of newborn genome screening based on insights from the NC NEXUS and BabySeq projects – two studies conducted in the US that aimed to explore the use of genomic sequencing in newborns in identifying actionable conditions and assess its impact on health outcomes. The authors highlight that the findings from these projects often identified specific risks that were difficult to quantify and required resource-intensive monitoring, rather than offering straightforward diagnoses with actionable treatments (Horton and Lucassen 2023). The added costs of repeated investigations and regular reviews over the lifetime of these patients – who may never develop these conditions – will significantly impact the NHS and should be appropriately addressed prior to embarking on a study of this scale.

Another important aspect of the NGP is the nature of informed consent. Parents will have to sign a consent form on behalf of their baby. It is therefore vital to determine how information on the 223 genetic diseases will be presented to parents and at what point in time will it be presented to ensure consent is informed. Information of this significance should be delivered by trained professionals in the appropriate settings and at an appropriate time, with both parents being present for informed consent (Science Media Center 2022). Indeed, both UKNSC and EURORDIS agree that whenever new programmes are piloted in the UK, all stakeholders should be involved in the planning of and implementation of the project, including designing and field-testing information and educational materials about the conditions included in screening programmes, the tests, and the subsequent treatment pathways, with the relevant stakeholders, modifying this information based on their feedback (Genomics England 2021; European Organisation for Rare Diseases 2021). This is considered essential for efficient implementation of the programme and to enable parents to make informed decisions about NBS, or in this case, the NGP.

## Discussion

**Fig. 2:**
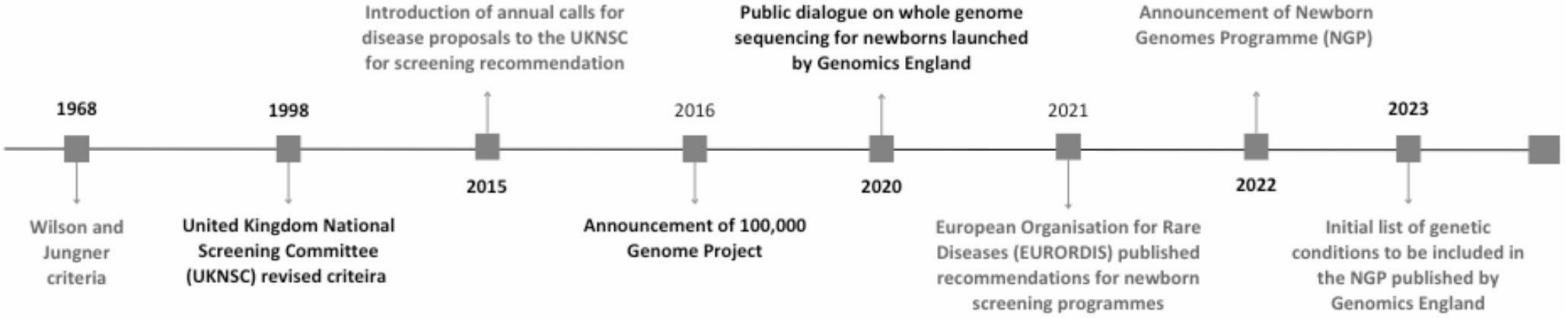
Key points in the evolution of newborn screening policy in the United Kingdom

Despite the fact that the UKNSC has promoted itself as having the most robust NBS programme globally, we question whether this has been in the interests of the patients, carers, and their physicians. The rigid adherence to the 1998 screening criteria created by the UKNSC has held back the diagnosis of rare diseases in many newborns, by restricting NBS to only nine diseases up to 2022. In certain cases, this may have resulted in irreversible disease progression e.g. hearing loss due to BD, in others, the stress and expense associated with the diagnostic odyssey and lack of timely access to support groups may have severely impacted the quality-of-life of many patients and their families. A summary of key points in the evolution of NBS policy in the UK is provided in Fig. 2. To note, in contrast to the UK, many countries collect newborn screening samples within the first 24–48 h of life to ensure timely detection of potentially serious conditions such that could manifest within the first week of life. While the UK approach may help reduce false-positive results for certain conditions, it also raises concerns regarding potential delays in diagnosing time-critical disorders such as Maple Syrup Urine Disease (MSUD) and Congenital Adrenal Hyperplasia (CAH) that require urgent intervention, as results can take six weeks to become available (Therrell et al. 2024). While these are legitimate concerns, they fall outside the scope of this paper.

Examination of the reasons given by the UKNSC for not recommending specific conditions highlights that it would be almost impossible to provide all the evidence required to meet the 20 criteria set in 1998, especially when the government does not provide funding to carry out the necessary research to address all the criteria. Some of the listed criteria are unlikely to be met for many rare diseases, other criteria prevent screening for diseases that may not be treatable but are actionable. The UKNSC has thus far adopted an algorithmic decision-making process. As such consistency is ensured, but many morally relevant factors are excluded. Indeed, an algorithmic decision-making process does not take into account many important moral arguments (Friesen et al. 2019). Instead, a discretionary decision-making process takes into account complex and multi-faceted factors and includes patient and carer voices and allows for certain inconsistencies in the process under certain circumstances. This is akin to the justice system, whereby sentencing takes into account many different factors. Having an inflexible decision-making process has led to the point where decisions are being reached that seem unreasonable to experts and patients. Indeed, adopting this process resulted in no new diseases being approved for NBS in UK between 2015 and 2022, putting the UK NBS dramatically behind other countries in the Global North.

Similar to the UK, several countries have initiated pilot studies integrating genome sequencing into NBS programmes. The United States (BabySeq, GUARDIAN), Australia (BabyScreen+), and Belgium (Baby Detect) have all introduced genomic screening pilots with varying degrees of flexibility in condition selection, expanding beyond conditions historically included in biochemical panels. Comparative analyses show substantial variation in gene and disease selection across countries, highlighting a lack of consensus on which conditions should be included in genomic sequencing pilot studies (Betzler et al. 2024). Nevertheless, there is a growing international trend toward less stringent inclusion criteria for genome sequencing programmes in comparison to biochemical assays, though this divergence is more pronounced in the UK. The lack of progress in NBS creates an anomalous position when Genomics England is just about to commence screening for over 200 rare genetic diseases in 100,000 newborns enlisted into their research study. Only four ethical principles need to be met for inclusion in the NGP, as compared to 20 screening criteria for the NBS. This raises the possibility that conditions listed in Table 2, and previously rejected by UKNSC, could be reviewed using the new NGP ethical criteria and not be rejected. This, in turn, prompts the question of whether the UKNSC should automatically reassess these diseases for inclusion in the broader NBS programme. Indeed, BD which was rejected for NBS by UKNSC three times over 10 years, is one of the diseases that will be screened for in the NGP, making it challenging for the UKNSC to justify this position, given their involvement in both the NBS and NGP.

Communications to the public from the Government and Genomics England have consistently implied that NGP is a pilot study that could ultimately extend the NBS programme and that the 200 plus diseases that will be tested for are essentially treatable (Parry 2023; Queen Mary University of London 2022). This raises a number of ethical concerns. Firstly, the diseases are being differentially described as either treatable or actionable dependent on the communication. This is likely to lead to confusion with the general public, who may not appreciate the critical difference in these terms. The inclusion of actionable diseases is at odds with the principles of the UKNSC, as laid out above. However, from the list of 223 genetic diseases published, it is clear that some will be actionable and not treatable and that some of the treatments involve gene editing and bone marrow transplants which may not be available within the time frame of the programme (Bick et al. 2021). In most countries as well as the UK, routine NBS is an opt-out process—parents do not need to actively consent, as it is considered a public health initiative focused on serious, treatable conditions (Horton and Lucassen 2023). Genomic sequencing pilots, however, require explicit informed consent. This need for informed consent should shape the disease selection process as researchers must justify which conditions are included in a way that parents will find acceptable. The terms “actionable” and “treatable” conditions should be defined, but parental perceptions of ‘actionability’ may still differ. Evidence maps for each disease should therefore be publicly available to justify inclusion, particularly for actionable diseases. Parents should also be informed during the consent process of the different treatment options for each disease to be tested. Secondly the communications suggest that the primary goal of the NGP is to expand the NBS programme (Genomics England 2023c). If this was the primary driver, as noted by others, the UK could simply extend the existing biochemical analysis of blood spots to nationally screen, at a low cost, for up to 35 rare genetic diseases, as other countries in the Global North are currently doing (Commonwealth of Australia - Department of Health and Aged Care 2023; National Screening Unit 2014).

It is important to appreciate the current relative costs of whole genome sequencing (£1050 / baby) versus biochemical analysis of blood spots (59p / baby) (Bessey et al. 2020). The cost of the NGP, funded by the Government is £105 million (United Kindgom Government 2022a). Given approximately 700,000 babies are born in the UK annually, the cost of increasing biochemical analysis would be £413,000 a year, versus £735 million a year for genomic screening (Bessey et al. 2020). If genomic screening became a standard screening method it would require a serious commitment of funding from the NHS, a system that is currently under extreme financial strain.

It is evident that the genetic diseases included in the NGP will not need to fulfil the 20 screening criteria as set out in 1998 by the UKNSC. Yet, it remains unclear how the UKNSC justifies this radical shift in their decision-making process or how Genomics England’s comparatively light-touch approach will influence the outcome. It is possible such shifts will lead to a loss in public confidence and trust in the UKNSC and its processes. Publication of the evidence maps, such that scientists and parents can see the decision-making process and be made aware of the treatment options for each of the 223 genetic diseases, would increase public confidence. Furthermore, justification as to why £105 million is to be spent on NGP is required, particularly when it would cost much less to extend the current NBS programme by MS/MS, or to extend the successful targeted genetic screening programme of critically ill babies and children as established in Wales, to other nations in the UK. Alternatively, if the primary driver for the NGP is to create a World-leading data resource that will drive research into genetic diseases and improve healthcare outcomes for the population this should be clarified in all communications for the general public. Open and transparent communication that the NGP is a research project and not ‘an extension of NBS’, as is implied on the website, would increase public understanding of the project, allow for more informed public engagement, and appropriately manage expectations.

## Data Availability

No datasets were generated or analysed during the current study.

## References

[CR1] All Wales Medical Genomics Service (2019) Wales Infants’ & Children’s Genome Service Available at: https://medicalgenomicswales.co.uk/index.php/health-professional-information/wings

[CR2] Bell J (2019) Drug companies pay for exclusive access to UK genetic data. Available at: https://www.biopharmadive.com/news/uk-biobank-genome-sequencing-investment-amgen-astrazeneca-johnson-gsk/562709/. Accessed 2024.

[CR3] Bessey A, Chilcott J, Pandor A et al (2020) The Cost-Effectiveness of expanding the UK newborn bloodspot screening programme to include five additional inborn errors of metabolism. Int J Neonatal Screen 6(4) 10.3390/ijns6040093 PMC7711627 33233828

[CR4] Betzler IR, Hempel M, Mutze U et al (2024) Comparative analysis of gene and disease selection in genomic newborn screening studies. J Inherit Metab Dis 47(5):945–970 38757337 10.1002/jimd.12750

[CR5] Bick D, Bick SL, Dimmock DP et al (2021) An online compendium of treatable genetic disorders. Am J Med Genet C Semin Med Genet 187(1):48–54 33350578 10.1002/ajmg.c.31874 PMC7986124

[CR6] Callaway E (2023) World’s biggest set of human genome sequences opens to scientists. Nature 624(7990):16–17 38036674 10.1038/d41586-023-03763-3

[CR7] Carlie Driscoll C, McPherson B (2010) Newborn screening systems: the complete perspective

[CR8] Chedrawi AK, Ali A, Al Hassnan ZN et al (2008) Profound biotinidase deficiency in a child with predominantly spinal cord disease. J Child Neurol 23(9):1043–1048 18645204 10.1177/0883073808318062

[CR9] Chung CCY, Leung GKC, Mak CCY et al (2020) Rapid whole-exome sequencing facilitates precision medicine in paediatric rare disease patients and reduces healthcare costs. Lancet Reg Health West Pac 1:100001 34327338 10.1016/j.lanwpc.2020.100001 PMC8315561

[CR10] Commonwealth of Australia - Department of Health and Aged Care (2023) Newborn bloodspot screening. Available at: https://www.health.gov.au/our-work/newborn-bloodspot-screening

[CR11] Costello Medical (2021) Newborn screening for biotinidase deficiency: an evidence map on screening for biotinidase deficiency for the UK National screening committee. Reportno. Report number|,| number|, date. Institution|, Place Published|

[CR12] Dahiphale R, Jain S, Agrawal M (2008) Biotinidase deficiency. Indian Pediatr 45(9):777–779 18820388

[CR13] Davies CS (2016) Annual Report of the Chief Medical Officer 2016. Reportno. Report Number|, Date. Place Published|: Institution|

[CR14] Delaye J, Cacciatore P, Kole A (2022) Valuing the burden and impact of rare diseases: A scoping review. Front Pharmacol 13:914338 35754469 10.3389/fphar.2022.914338 PMC9213803

[CR15] Dimmock DP, Clark MM, Gaughran M et al (2020) An RCT of rapid genomic sequencing among seriously ill infants results in high clinical utility, changes in management, and low perceived harm. Am J Hum Genet 107(5):942–952 33157007 10.1016/j.ajhg.2020.10.003 PMC7675004

[CR16] Downing M, Pollitt R (2008) Newborn bloodspot screening in the UK–past, present and future. Ann Clin Biochem 45(Pt 1):11–17 18275669 10.1258/acb.2007.007127

[CR17] European Organisation for Rare Diseases (2017) Juggling care and daily life: the balancing act of the rare disease community. Reportno. Report number|,| number|, date. Institution|, Place Published|

[CR18] European Organisation for Rare Diseases (2021) Key principles for newborn screening. Reportno. Report number|,| number|, date. Institution|, Place Published|

[CR19] Ferreira CR (2019) The burden of rare diseases. Am J Med Genet A 179(6):885–892 30883013 10.1002/ajmg.a.61124

[CR20] Friesen P, Yusof ANM, Sheehan M (2019) Should the decisions of institutional review boards be consistent?? Ethics Hum Res 41(4):2–14 31336039 10.1002/eahr.500022

[CR21] Genetic Alliance UK (2019) Fixing the present Building for the Future - Newborn screening for rare conditions. Reportno. Report number|,| number|, date. Institution|, Place Published|

[CR22] Genomics England (2021) New public dialogue finds support for the use of whole genome sequencing in newborn screening – providing that the right safeguards and resources are in place 8 Jul 2021. Available at: https://www.genomicsengland.co.uk/news/public-dialogue-genomics-newborn-screening

[CR23] Genomics England (2023a) Conditions List Available at: https://www.genomicsengland.co.uk/initiatives/newborns/choosing-conditions/conditions-list-generation-study

[CR24] Genomics England (2023b) Newborn Genomes Programme Available at: https://www.genomicsengland.co.uk/initiatives/newborns/choosing-conditions?chapter=principle-d. Accessed 2023.

[CR26] Genomics England (2023d) Newborn Genomes Programme - Ethics Available at: https://www.genomicsengland.co.uk/initiatives/newborns/ethics. Accessed 30/11/2023.

[CR27] Genomics England (2025) The story behind us and The 100,000 Genomes Project. Available at: https://www.genomicsengland.co.uk/about-us/origins

[CR25] Genomics England (2023c) Newborn Genomes Programme. Available at: https://www.genomicsengland.co.uk/initiatives/newborns

[CR28] Goldenberg AJ, Comeau AM, Grosse SD et al (2016) Evaluating harms in the assessment of net benefit: A framework for newborn screening condition review. Matern Child Health J 20(3):693–700 26833040 10.1007/s10995-015-1869-9 PMC4819963

[CR29] Group NIS, Krantz ID, Medne L et al (2021) Effect of Whole-Genome sequencing on the clinical management of acutely ill infants with suspected genetic disease: A randomized clinical trial. JAMA Pediatr 175(12):1218–1226 34570182 10.1001/jamapediatrics.2021.3496 PMC8477301

[CR30] Health Resources and Services Administration - Advisory Committee on Heritable Disroders in Newborns and Children (2023) Recommended Uniform Screening Panel

[CR31] Hopkins Van Mil (2021) Implications of whole genome sequencing for newborn screening. Reportno. Report number|,| number|, date. Institution|, Place Published|

[CR32] Horton R, Lucassen A (2023) Ethical issues Raised by new genomic technologies: the case study of newborn genome screening. Camb Prism Precis Med 1:e2 38550936 10.1017/pcm.2022.2 PMC10953737

[CR33] Jezkova J, Shaw S, Taverner NV et al (2022) Rapid genome sequencing for pediatrics. Hum Mutat 43(11):1507–1518 36086948 10.1002/humu.24466 PMC9826377

[CR34] Joynson C (2021) Embedding ethics at the UK National Screening Committee. Available at: https://phescreening.blog.gov.uk/2021/03/23/embedding-ethics-at-the-uk-national-screening-committee/

[CR35] Jungner G, Wilson JMG (1966) Principles and Practice of Screening for Disease World Health Organization, Geneva, 1968

[CR36] Kemper AR, Green NS, Calonge N et al (2014) Decision-making process for conditions nominated to the recommended uniform screening panel: statement of the US department of health and human services Secretary’s advisory committee on heritable disorders in newborns and children. Genet Med 16(2):183–187 23907646 10.1038/gim.2013.98

[CR37] Liu S, Zhang Y, Deng Z et al (2023) Delayed Biotin Therapy in a Child with Atypical Profound Biotinidase Deficiency: Late Arrival of the Truth and a Lesson Worth Thinking. Int J Mol Sci 24(12) 10.3390/ijms241210239 PMC10299609 37373384

[CR38] Loeber JG, Platis D, Zetterstrom RH et al (2021) Neonatal Screening in Europe Revisited: An ISNS Perspective on the Current State and Developments Since 2010. Int J Neonatal Screen 7(1) 10.3390/ijns7010015 PMC8006225 33808002

[CR39] Lombardo S, Seedat F, Elliman D et al (2023) Policy-making and implementation for newborn bloodspot screening in Europe: a comparison between EURORDIS principles and UK practice. Lancet Reg Health Eur 33:100714 37954001 10.1016/j.lanepe.2023.100714 PMC10636270

[CR40] Mackie A (2023) Evaluation of NHS newborn screening for SCID extended to March 2024. Available at: https://nationalscreening.blog.gov.uk/2023/06/13/evaluation-of-nhs-newborn-screening-for-scid-extended-to-march-2024/#:~:text=The%20NHS%20invites%20and%20screens,were%20referred%20with%20abnormal%20results. Accessed 30/11/2023

[CR41] Mestek-Boukhibar L, Clement E, Jones WD et al (2018) Rapid paediatric sequencing (RaPS): comprehensive real-life workflow for rapid diagnosis of critically ill children. J Med Genet 55(11):721–728 30049826 10.1136/jmedgenet-2018-105396 PMC6252361

[CR42] Murch OJJ, Halstead J et al (2021) 1165 The Wales infants’ and children’s genome service’ (WINGS): diagnostic rapid whole genome sequencing for unwell children with a suspected rare genetic diagnosis. Arch Dis Child 106

[CR43] National Screening Unit (2014) Newborn metabolic screening programme - heel prick test Available at: https://www.health.govt.nz/your-health/pregnancy-and-kids/first-year/first-6-weeks/health-checks-first-6-weeks/newborn-screening-tests/newborn-metabolic-screening

[CR44] Nguengang Wakap S, Lambert DM, Olry A et al (2020) Estimating cumulative point prevalence of rare diseases: analysis of the Orphanet database. Eur J Hum Genet 28(2):165–173 31527858 10.1038/s41431-019-0508-0 PMC6974615

[CR45] Online Mendelian Inheritance in Man (OMIM) (2023) Biotinidase Deficiency Online Mendelian Inheritance in Man

[CR46] Page T (2023) 100,000 newborn babies will have their genomes sequenced in the UK. It could have big implications for child medicine. CNN Health https://edition.cnn.com/2023/03/19/health/newborn-genomes-programme-uk-genomics-scn-spc-intl/index.html

[CR47] Parry V (2023) Which conditions will we look for initially in the generation. Study? Genomics England Website

[CR48] Pichini A, Ahmed A, Patch C et al (2022) Developing a National newborn genomes program: an approach driven by ethics, engagement and Co-design. Front Genet 13:866168 35711926 10.3389/fgene.2022.866168 PMC9195613

[CR49] Queen Mary University of London (2022) UK Government launches Newborn Genomes Programme

[CR50] Salova M, Bachman S (2025) Newborn screening: landscape and rare disease developments. Available at: https://avalere.com/insights/newborn-screening-landscape-and-rare-disease-developments

[CR51] Science Media Center (2022) expert reaction to launch of the newborn whole genome sequencing pilot programme. Available at: https://www.sciencemediacentre.org/expert-reaction-to-launch-of-the-newborn-whole-genome-sequencing-pilot-programme/

[CR52] Seedat FLS (2022) Meeting the challenges of rare diseases in NHS newborn blood spot screening. Available at: https://nationalscreening.blog.gov.uk/2022/07/19/meeting-the-challenges-of-rare-diseases-in-nhs-newborn-blood-spot-screening/. Accessed 2023.

[CR53] Szymanska E, Sredzinska M, Lugowska A et al (2015) Outcomes of oral biotin treatment in patients with biotinidase deficiency - Twenty years follow-up. Mol Genet Metab Rep 5:33–35 28649539 10.1016/j.ymgmr.2015.09.004 PMC5471405

[CR54] The Lancet (2023) Genomic newborn screening: current concerns and challenges. Lancet 402(10398):265 37481265 10.1016/S0140-6736(23)01513-1

[CR55] Therrell BL, Padilla CD, Borrajo GJC et al (2024) Current status of newborn bloodspot screening worldwide 2024: A comprehensive review of recent activities (2020–2023). Int J Neonatal Screen 10(2) 10.3390/ijns10020038 PMC11203842 38920845

[CR56] UK Health Security Agency (2018) In: Why we need to count the people who have rare diseases. Available at: https://ukhsa.blog.gov.uk/2018/02/27/why-we-need-to-count-the-people-who-have-rare-diseases/. Accessed 2023.

[CR57] United Kindgom Government (2022a) Over £175 million for cutting-edge genomics research. Online

[CR66] United Kingdom National Screening Committee (2022) Criteria for a population screening programme. Available at: https://www.gov.uk/government/publications/evidence-review-criteria-national-screening-programmes/criteria-for-appraising-the-viability-effectiveness-and-appropriateness-of-a-screening-programme

[CR65] United Kingdom National Screening Committee (2021) 25 years on: how the UK NSC has transformed screening for the better. In: Available at: https://phescreening.blog.gov.uk/2021/07/30/25-years-on-how-the-uk-nsc-has-transformed-screening-for-the-better/. Accessed 2023.

[CR63] United Kingdom National Screening Committee (2019) Generation genome and the opportunities for screening programmes

[CR59] United Kingdom Government (2022) UK NSC blood spot task group (BSTG). Available at: https://www.gov.uk/government/groups/uk-nsc-blood-spot-task-group-bstg

[CR58] United Kindgom Government (2022b) UK National Screening Committee - About us. Available at: https://www.gov.uk/government/organisations/uk-national-screening-committee/about

[CR60] United Kingdom Government Department of Health (2013) The UK Strategy for Rare Diseases

[CR61] United Kingdom Government Department of Health (2023) England Rare Diseases Action Plan 2023: main report

[CR62] United Kingdom National Screening Committee (1998) National Screening Committee. First report of the National Screening Committee. In: Kingdom HDotU (ed)

[CR68] United Kingdom National Screening Committee (2023b) UK NSC Blood Spot Task Group July 2023 meeting summary. Available at: https://www.gov.uk/government/publications/uk-nsc-blood-spot-task-group-july-2023-meeting-summary

[CR64] United Kingdom National Screening Committee, UK National Screening Committee (UK NSC) (2020): Note of the meeting held on the 26 February 2020 at Park Plaza London Riverbank, 18 Albert Embankment, London, SE1 7TJ.

[CR67] United Kingdom National Screening Committee (2023a) UK NSC annual call: submitting a screening proposal

[CR69] Wolf B (1993) Biotinidase Deficiency. In: Adam MP, Mirzaa GM, Pagon RA, (eds) GeneReviews((R)). Seattle (WA) 20301497

[CR70] Wolf B (2017) Successful outcomes of older adolescents and adults with profound biotinidase deficiency identified by newborn screening. Genet Med 19(4):396–402 27657684 10.1038/gim.2016.135

[CR71] Wolf B, Heard GS, Jefferson LG et al (1985) Clinical findings in four children with biotinidase deficiency detected through a statewide neonatal screening program. N Engl J Med 313(1):16–19 4000223 10.1056/NEJM198507043130104

[CR72] Yang Y, Yang JY, Chen XJ (2020) Biotinidase deficiency characterized by skin and hair findings. Clin Dermatol 38(4):477–483 32972606 10.1016/j.clindermatol.2020.03.004

